# Microstructure and Mechanical Properties of Ti-6Al-4V Welds Produced with Different Processes

**DOI:** 10.3390/ma17040782

**Published:** 2024-02-06

**Authors:** Sakari Tolvanen, Robert Pederson, Uta Klement

**Affiliations:** 1 Department of Industrial and Materials Science, Chalmers University of Technology, SE-41296 Gothenburg, Sweden; sakari.tolvanen@gknaerospace.com (S.T.); uta.klement@chalmers.se (U.K.); 2 Department of Engineering Science, University West, SE-46186 Trollhattan, Sweden

**Keywords:** titanium alloys, welding, defects, porosity, microstructure, fatigue

## Abstract

The effect of defects and microstructure on the mechanical properties of Ti-6Al-4V welds produced by tungsten inert gas welding; plasma arc welding; electron beam welding; and laser beam welding was studied in the present work. The mechanical properties of different weld types were evaluated with respect to micro hardness; yield strength; ultimate tensile strength; ductility; and fatigue at room temperature and at elevated temperatures (200 °C and 250 °C). Metallographic investigation was carried out to characterize the microstructures of different weld types, and fractographic investigation was conducted to relate the effect of defects on fatigue performance. Electron and laser beam welding produced welds with finer microstructure, higher tensile ductility, and better fatigue performance than tungsten inert gas welding and plasma arc welding. Large pores, and pores located close to the specimen surface, were found to be most detrimental to fatigue life.

## 1. Introduction

Titanium alloys used in fabricated aerospace structures require joints of high integrity to meet design requirements. Tungsten inert gas welding (TIG), plasma arc welding (PAW), laser beam welding (LBW), and electron beam welding (EBW) are all processes capable of creating high quality fusion-welded joints in titanium alloys. The highly concentrated energy input in high energy beam welding processes permits the generation of a keyhole, which allows deep penetration with low total heat input. Microstructural changes are confined to narrow fusion and heat affected zones (HAZ), and residual stresses are relatively low, which produces welds with appropriate mechanical and fatigue properties [1]. TIG and PAW offer the potential to achieve welds of equal quality to EBW or LBW at much lower capital costs. While the higher heat input in arc welding processes produces wider weld zones with coarser microstructure, it has been shown that TIG welds can have comparable mechanical properties to EBWs in both cast and wrought base materials [2].

The microstructures formed in α + β titanium alloys during continuous cooling are complex. The cooling rate affects the transformed β microstructure in the fusion zone and in the HAZ. At cooling rates faster than 410 K/s, transformation occurs completely through martensitic transformation, forming acicular α’. At lower cooling rates, this transformation is gradually replaced by diffusion-controlled Widmanstätten α-formation [3]. An intermediate cooling rate favors basketweave type of structures, whereas a lower cooling rate mainly leads to a colony type of microstructure consisting of several aligned and parallel α plates that together forms a “colony” [4]. In the alloy Ti-6Al-4V (Ti-64), the as-welded microstructure in arc welding typically consists of a combination of martensitic α’ and fine diffusional transformed α plates. In EBW and LBW, the as-welded microstructure can be entirely martensitic [1,5]. Post weld heat treatments (PWHT) are applied to reduce residual stresses, stabilize and homogenize the weld zone microstructure, and to improve ductility [6]. Microstructural changes occur during post weld heat treatments as the metastable α’ decomposes to equilibrium α and β, and the microstructure coarsens [5,7,8,9].

Low ductility in the fusion zone is a typical feature in α + β titanium alloy welds, and has been attributed to a large prior-β grain size and an acicular intragranural microstructure [5,10,11]. According to Lütjering et al. [11], prior-β grain size has a strong influence on ductility. Sundaresan et al. [12] showed that the ductility of Ti-6Al-4V and Ti-6Al-2Sn-4Zr-2Mo welds could be improved by using pulsing current to reduce prior-β grain size. Poor ductility of acicular microstructures has also been explained by the large area of α-β interfaces per unit volume, because cracks have been observed to nucleate at these interfaces [11]. The low cycle fatigue (LCF) performance of Ti alloy welds is affected by the microstructure of the welds, presence of defects, residual stresses, and loading conditions. According to Lütjering [11], the LCF strength of a material is a result of two contributing factors: its resistance to crack nucleation and its resistance to microcrack propagation. In α + β titanium alloys both factors increase with the increasing cooling rate from the β phase field. The influence of defects and porosity on crack initiation and fatigue life have been examined by both experimental observations and through theoretical modelling [13,14,15,16,17,18,19,20]. Size and location of the pores are important. It has been shown that pores close to or at the surface cause the highest stress concentration.

The welds produced with EBW, LBW, TIG, and PAW have different microstructures, and different populations of defects in terms of their size and distribution. The objective of this paper is to study the influence of these aspects on the mechanical properties of welds under different testing conditions. The microstructure and defect populations in the different welds have been characterized by metallography, X-ray microscopy, and fractography. The mechanical testing included harness measurement, tensile testing, and low cycle fatigue.

## 2. Materials and Methods

A 4 mm thick Ti-6Al-4V sheet material (AMS4911, RTI International Metals, Pittsburgh, PA, USA) was used as the base material to produce welded samples with TIG, PAW, EBW, and LBW. Welding parameters optimized for 4 mm material thickness were used for each process. EBW, LBW and PAW welds were produced autogenously, and for TIG welds a filler wire was used (AMS4954H). The chemical compositions of the materials are given in Table 1. After welding, a post weld heat treatment at 704 °C for 2 h was applied.

**Figure 1:**
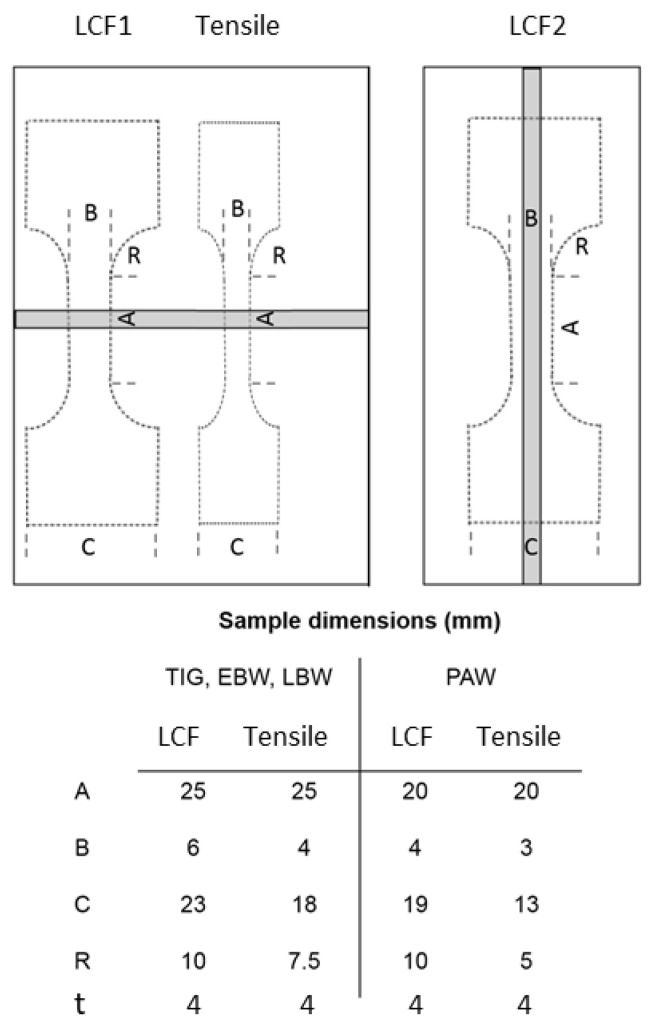
Schematic drawings and dimensions of the samples used for mechanical testing. Tensile and LCF type 1 specimens were perpendicular to the weld zone, and the LCF type 2 specimen was parallel to the weld zone.

The external weld geometry was machined off from the mechanical test specimens. This was conducted in order to minimize the influence of the weld geometry in order to be able to study specifically the effect of microstructure and weld defects on the mechanical properties. The strain-controlled tensile testing for the base material and for the welds was performed according to ASTM E8 [21] at room temperature, and according to ASTM E21 [22] at 250 °C. The tensile testing was performed in transverse direction to the weld. Load controlled fatigue testing was performed according to ASTM E466 [23] at room temperature, and at 250 °C in air atmosphere, with stress ratio R = 0. In addition, a number of LBW samples were not polished after welding in order to investigate the influence of external weld geometry on fatigue properties. These samples were tested at 200 °C. Fatigue test samples were prepared in both transverse and longitudinal directions to the weld. The number of cycles to fracture and the total stress range were recorded during each test, and plotted in a diagram to show the low cycle fatigue strength. The dimensions and schematic drawings of the samples used for mechanical testing are shown in Figure 1.

An optical microscope and a LEO Gemini 1550 FEG scanning electron microscope were used to examine the microstructure in the fusion zone (FZ). Sample preparation was conducted using conventional metallographic techniques for titanium alloys, involving grinding, polishing, and etching using Kroll’s etchant. The microhardness measurement was performed using a Shimadzu HMV-2000 machine (Shimadzu, Kyoto, Japan) with a load of 500 g. Post-test fractographic analysis of tensile test samples and LCF samples were performed using a scanning electron microscope. Size and location of pores on the fracture surfaces of LCF samples were determined by measuring the shortest distance to the sample surface.

The oxygen content of TIG and EBW welds in comparison to the base material was measured with a JEOL JXA-8500F (JEOL, Tokyo, Japan) electron probe micro analyzer (EPMA). Qualitative line scans across the welds were performed using the instrument parameters of 10 kV acceleration voltage, 30 nA beam current with a 100 µm step size, and a 5 µm defocused beam.

X-ray microscopic (XRM, similar to XCT) investigations were performed on selected LCF specimens using Carl Zeiss Xradia 520 Versa equipment (Carl Zeiss, Jena, Germany). The scan volume was 3 mm × 5 mm × 8 mm with 4 µm voxel size, and 3 mm × 5 mm × 1 mm with 1 µm voxel size.

## 3. Results and Discussion

### 3.1. Microstructure

**Figure 2:**
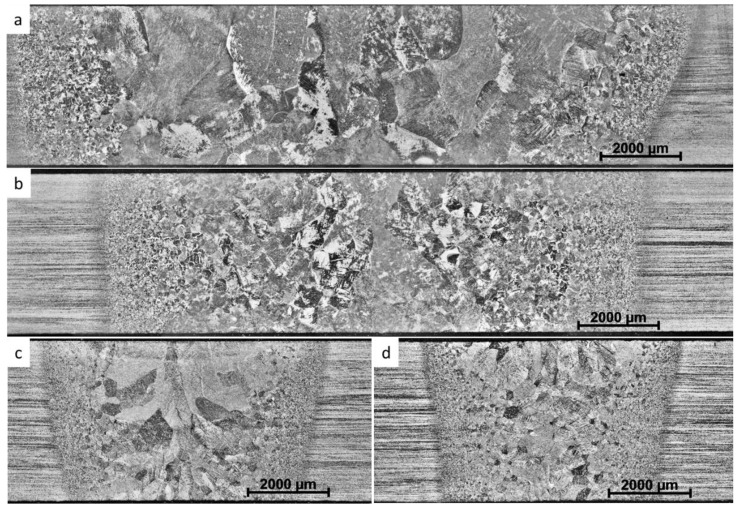
Optical micrographs showing the weld cross sections for (**a**) TIG, (**b**) PAW, (**c**) EBW, and (**d**) LBW welds in 4 mm thick Ti-64 sheet material.

Optical micrographs of the studied post weld heat-treated welds are shown in Figure 2. The heat-affected zones (HAZs) are characterized by prior-β grains that gradually become coarser towards the fusion zones. In general, the width of the welds was slightly wider at the top than at the bottom. The dimensions of all examined welds and the average hardness of fusion zones are summarized and shown in Table 2. The TIG weld was widest and contained the largest prior-β grains. The fusion zone (FZ) and prior-β grain size was smaller in PAWs than in TIG welds. Welds produced by EBW and LBW were considerably narrower. LBW had the smallest weld zone and smallest prior-β grain size. Also, the prior-β grains in LBW were more equiaxed as compared to the other welds where prior-β grains are elongated.

**Figure 3:**
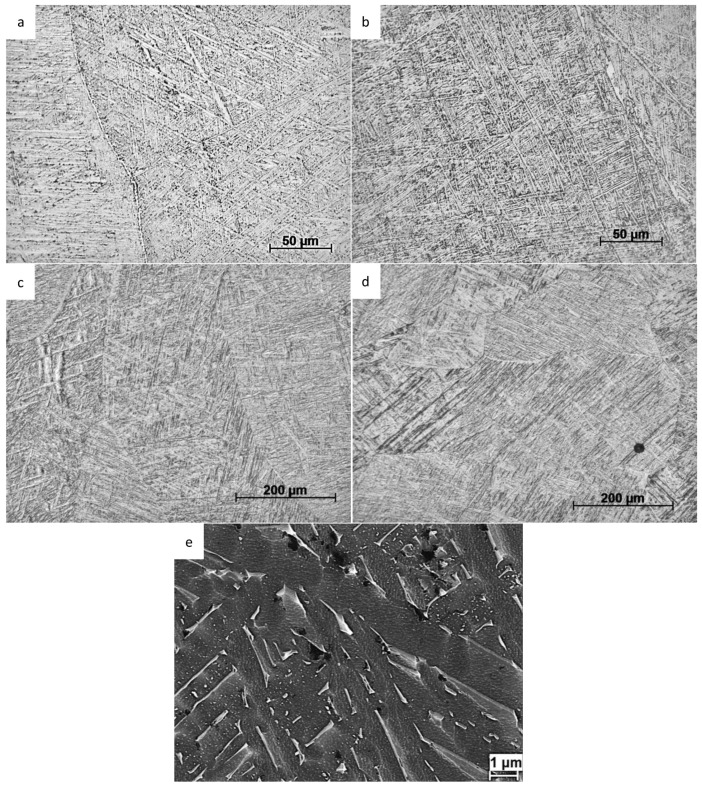
Optical micrographs of (**a**) TIG, (**b**) PAW, (**c**) EBW, and (**d**) LBW welds, and (**e**) SEM micrograph of PAW weld. The different magnifications can be noticed.

Figure 3 shows typical microstructures inside prior-β grains in the fusion zones of each type of weld after post weld heat treatment. The microstructure of all the welds consisted of fine α laths separated by thin layers of β phase, as shown in Figure 3e in the PAW weld. Inside prior-β grains in TIG and PAW, the microstructure consisted of both colony and basketweave types of microstructures. In the TIG welds, the fraction of colony structure was higher than in the other welds. This is because of the higher heat input and slower cooling rate in TIG welds as compared with the other welding processes, which promotes the transformation into a colony type of structure [4]. In EBW and LBW the α laths were more acicular and they formed a very fine basketweave type of microstructure. In EBW and LBW, grain boundary-α was observed in a few prior-β grain boundaries. In TIG and PAW, the grain boundary-α was more continuous and thicker. The average α lath spacing in EBW and LBW were estimated to be 0.9 ± 0.1 µm, and in TIG and PAW the corresponding α lath spacing were estimated to be 1.2 ± 0.1 µm. These values were based on 10 optical micrographs investigated for each weld. In addition, porosity was noticeable in the LBW micrographs.

**Figure 4:**
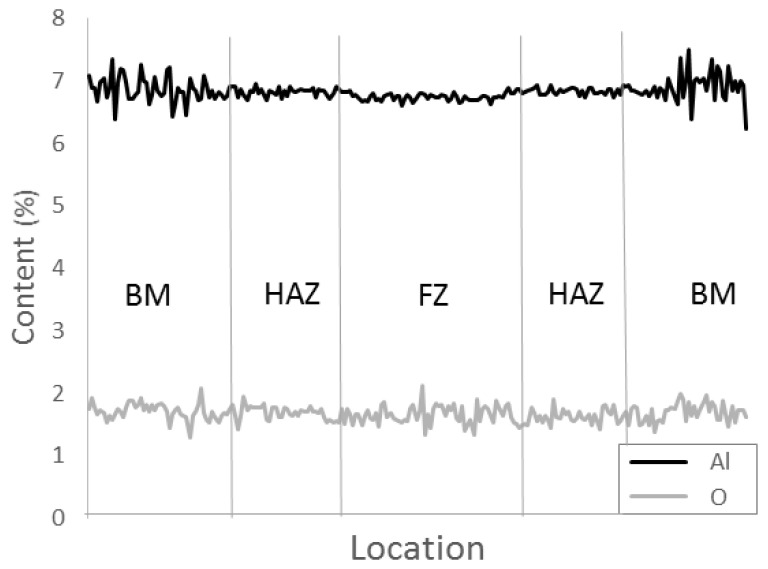
EPMA line scan across a TIG weld showing the distribution of aluminium and oxygen across the weld, i.e., in the base material, the heat affected zone, and the fusion zone.

In Figure 4, a qualitative electron probe micro analysis (EPMA) line scan across a TIG weld is shown. The absolute values are not correct; however, the relative variation between base material and the weld zone are correct. The oxygen content in the base material was 0.12 wt.%. The EPMA results revealed no difference in oxygen content between the base material and weld zones. A small drop in aluminium was observed in the fusion zone. Aluminium has a lower boiling point than titanium and vanadium, which is why it can vaporize more easily during the welding process. An EPMA line scan across an EBW showed similar behaviour.

### 3.2. Tensile Properties and Microhardness

**Figure 5:**
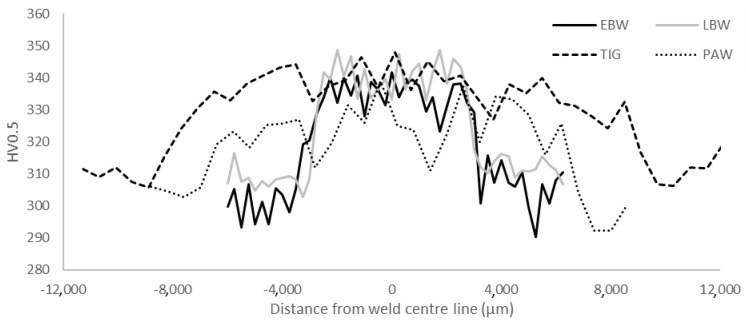
Microhardness profiles across welds produced by EBW, LBW, TIG and PAW after post weld heat treatment.

Microhardness profiles across the different weld cross-sections are summarized in Figure 5. In general, all four types of welds showed a similar trend. The hardness was found to be lowest in the base material and increasing through the HAZ, with the maximum hardness located in the fusion zone. The average hardnesses of the weld fusion zones are shown in Table 2.

The results from tensile testing are given in Table 3. The base material had higher strength than the welds, despite the higher hardness of the welds. Welds produced by EBW and LBW had 3–5% higher strength in comparison to the TIG and PAW welds. Increasing temperature from RT to 250 °C reduced strength by approximately 20% in all cases.

The ductility of the welds was lower than the ductility of the base material. The reduction in area for the base material was 44% and the tensile elongation was 16.4%, when tested at room temperature. Of the welds, the LBW had the highest ductility, followed by the EBW and PAW welds. The TIG weld was found to have the lowest ductility with a tensile elongation of only 7%. At elevated temperature the ductility of the welds improved, but not to the level of the base material.

**Figure 6:**
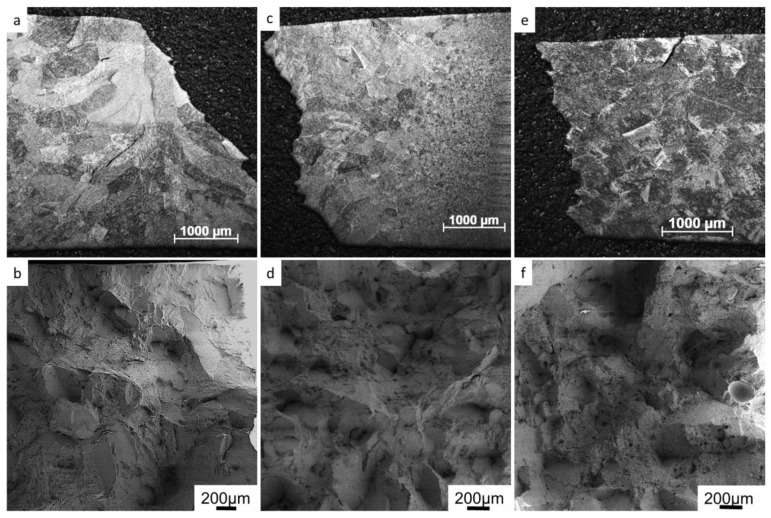
Room-temperature tensile tested weld samples. Etched side views and fracture surfaces of (**a**,**b**) EBW, (**c**,**d**) LBW, and (**e**,**f**) PAW.

In all welded tensile test specimens, the fracture was located in the weld zones. In Figure 6, etched side views and the fracture surfaces of tensile test welds tested at room temperature are shown. In all welds, the fracture was found to be a combination of transgranural and intergranular fracture. Moreover, cracks were also observed along prior-β grain boundaries, and through prior-β grains outside the main fracture. Areas of intergranular fracture appear as flat areas in fractographs in Figure 6b,d,f. The shape and size of these areas correspond to the prior-β grains in the microstructure. Dimples were found in all areas indicating fracture through coalesced micro voids.

### 3.3. Low Cycle Fatigue Properties

**Figure 7:**
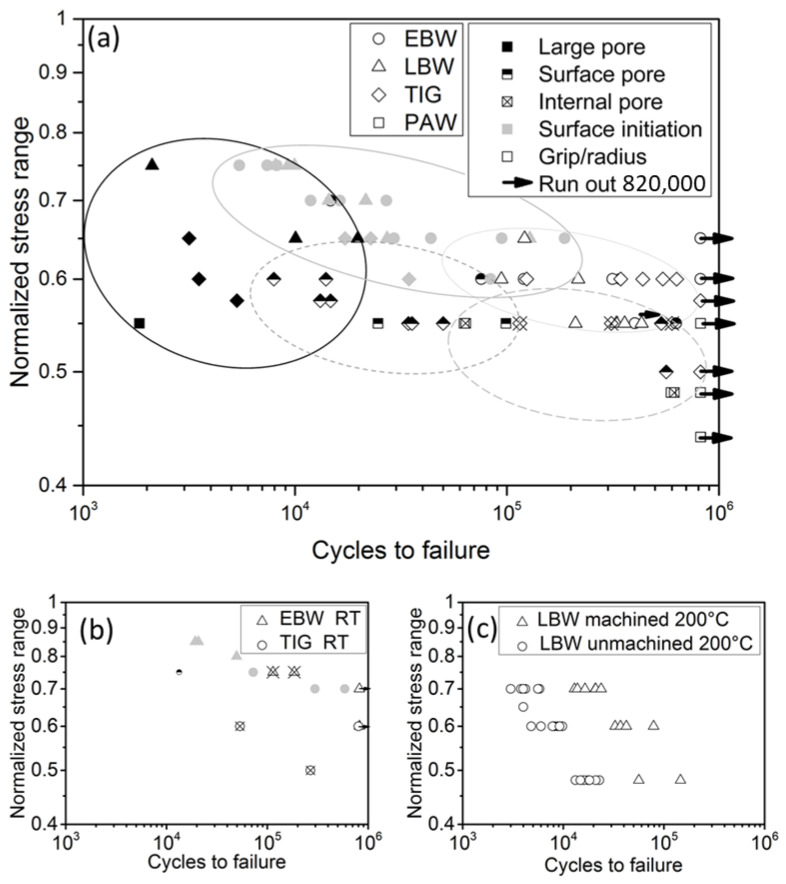
LCF results (**a**) for welds produced by TIG, PAW, EBW, and LBW at 250 °C; (**b**) EBW and TIG at room temperature; and (**c**) the effect of weld geometry in LBW. In (**a**,**b**), the shape of the symbol indicates the welding process, and the fill of the symbol indicates the type of initiation.

Results from low cycle fatigue testing at room temperature, and at 250 °C, are shown in Figure 7. The stress range values have been normalized with regard to the highest stress range applied during testing. Crack initiation sites have been categorized as *surface pores*, *internal pores*, *large pores* (>400 µm), and *surface initiation*. Each type is indicated by a unique symbol. Grip/radius samples failed outside the cage section of the test specimen, which is typically considered an invalid test. Here the specimens were included because they performed better than average. The testing direction was not found to have significant influence, and the results from the specimen types 1 and 2 (Figure 1) have been combined in Figure 7.

At 250 °C, welds produced by EBW and LBW were found to have superior LCF performance in comparison to the welds produced by TIG and PAW. EBW seems to have the best LCF performance and the smallest scatter of data. Most of the LBW samples have a similar fatigue life as the EBW samples, but the scatter of the data for the LBW samples is higher. The main reason for this was the significantly lower fatigue life in a few of the LBW samples, caused by large pores. The fatigue strength of welds produced by PAW and TIG was found to be similar to each other. However, because of the limited number of tested samples, these results should be interpreted as indicative rather than as conclusive.

In the LCF tests performed at room temperature (Figure 7b), the EBW and TIG welds had higher fatigue strength than at elevated temperature. The welds produced by EBW performed better and had considerably smaller scatter than the TIG welds.

**Figure 8:**
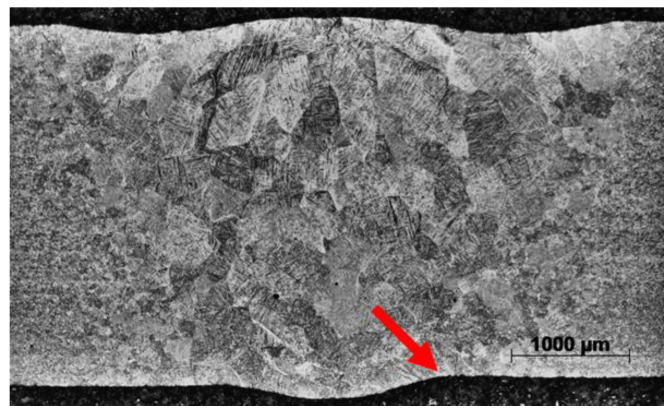
Optical micrograph of LBW showing the weld geometry. Typical crack initiation site at the weld toe on the root side indicated.

The LCF testing of polished and unpolished LBW specimens showed that the external weld geometry has a significant effect on the fatigue strength. The weld geometry of unpolished LBW is shown in Figure 8. Weld face underfill was found to be 80–100 µm, and root undercut 30–50 µm. As shown in Figure 7c, the machined samples had 5–10 times longer fatigue life compared with the samples with the external weld geometry retained. In the unpolished specimens, cracks always initiated at the weld toe on the root side and never at internal defects.

### 3.4. Fractographic Investigation

**Figure 9:**
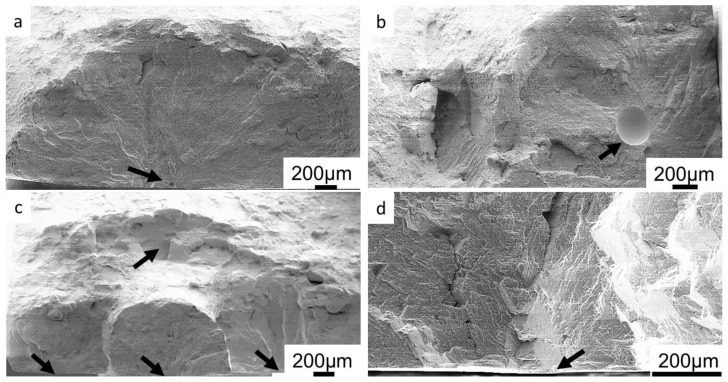
Fatigue fracture surfaces showing different types of crack initiation sites: (**a**) a small pore close to surface (EBW); (**b**) a large internal pore (TIG); (**c**) initiations at surface and at a cluster of pores (LBW); and (**d**) a surface initiation site with a facet (EBW).

Fractographic investigation of LCF samples with lower fatigue life mainly revealed two characteristics: (i) fatigue crack initiation at a large pore in the center of the sample, or (ii) fatigue crack initiation at a pore located close to the surface of the specimen. Fatigue specimens with long fatigue life typically had crack initiation at the surface of the sample. Fracture surfaces showing typical crack initiation sites and crack propagation areas around them are shown in Figure 9. Arrows indicate the crack initiation sites.

The pores and their size distribution found on the fracture surfaces of the samples tested at 250 °C are summarized in Table 4. In the first row, the total number of samples tested for each type of weld, as well as the number of samples that were eligible for fractographic investigation, is given. In the second row of Table 4, the total number of pores found on fracture surfaces are given, including those that did not initiate cracks. Specimens that fractured outside of the gage section, or that did not fail during the test, were disregarded from fractographic investigation. In the EBW samples, most of the fracture initiations occurred at the surface of the sample. Out of 28 EBW specimens investigated, 24 had crack initiation at the surface, 2 samples show crack initiation at a small pore close to the surface, and 2 samples at a small internal pore. Most LBW samples also had crack initiation at the surface, and in 3 samples the crack initiation occurred at large pores of approximately 650 µm in diameter. In LBW specimens, small pores (<200 µm) which did not initiate cracks were observed on the fracture surfaces. In one LBW sample, fatigue crack growth pattern was found around a cluster of pores (Figure 9c), but even in this sample the main contribution to the fatigue fracture came from cracks that initiated at the surface. For TIG samples, the average size of a pore initiating a crack was 212 μm. The largest and smallest crack-initiating pores found in TIG samples were 436 μm and 58 μm in diameter, respectively. For PAW samples, the average pore size initiating a crack was 113 μm, while the largest and smallest pores initiating a crack were found to be 182 μm and 68 μm in diameter, respectively. In addition, a lack of fusion (LOF) defect was found in one sample. Comparing the pore size distribution in different welds, it can be seen that the pores produced in EBW are the smallest, followed by PAW. LBW produced the largest pores and many small pores, which was confirmed via X-ray microscopic (XRM) investigation. The results of the XRM study are shown in a following chapter.

**Figure 10:**
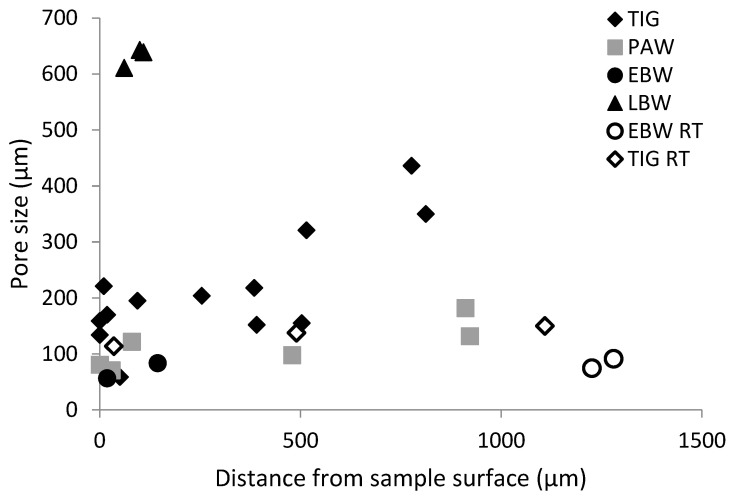
The sizes and location of pores initiating fatigue cracks.

In Figure 10, the size of pores which initiated a crack, as well as their distance to the sample surface, are shown. Approximately half of the pores that initiated cracks had a larger diameter than its respective distance to the surface.

Only pores close to the surface initiated cracks that led to failure in EBW and LBW samples, when tested at elevated temperature. In all other EBW and LBW samples, facets were found at crack initiation sites (e.g., Figure 9d). In TIG and PAW samples, internal pores were also found to initiate cracks.

At room temperature, the influence from internal pores seems to be more pronounced on fatigue performance. Small internal pores are found to initiate cracks at room temperature in EBW and TIG specimens. This was not observed in EBW specimens tested at 250 °C.

### 3.5. X-ray Microscopy

**Figure 11:**
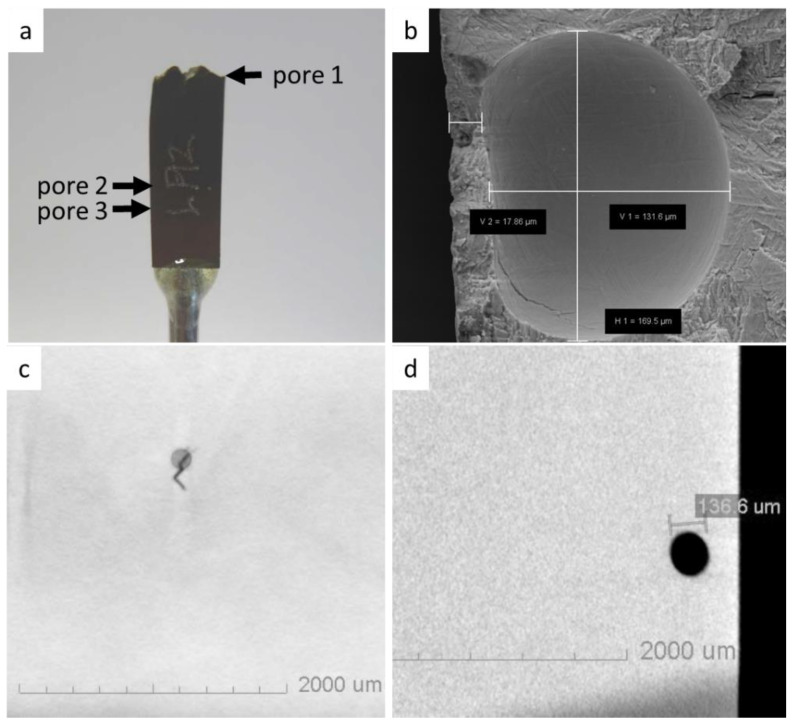
TIG welded LCF specimen investigated with XRM: (**a**) overview of the fractured LCF specimen; (**b**) SEM fractograph of pore 1, which initiated the fatigue crack that led to failure of the TIG welded LCF specimen; (**c**) XRM virtual slice of the surface-breaking pore 2, with a diameter of 136 µm, which initiated a 300–400 μm long microcrack; and (**d**) XRM virtual slice of pore 3 which did not initiate a crack.

X-ray microscopy scans were performed on 2 TIG and LBW LCF specimens (LCF Type 2). All the specimens were tested at a normalized stress range of 0.55. The results of the XRM scan of a TIG specimen that failed after 13,166 cycles are shown in Figure 11. Figure 11b shows a pore on the fracture surface, which initiated the crack leading to failure in the TIG welded sample. Figure 11c,d shows two 2D virtual slices from the XRM 3D scans of the same specimen. Pore 1, which caused the failure of the sample, was 169 µm in diameter. In the XRM scan in Figure 11c, a surface-breaking pore, pore 2, with a diameter of 136 µm was found, which initiated a crack of approximately 300–400 µm in length. A third pore, pore 3, with a diameter of 148 µm, was found located right below the fracture surface (Figure 11d). Pore 3 did not initiate a crack. The other TIG welded LCF specimen was discontinued after 820,000 cycles since no failure occurred. The XRM scan of this specimen revealed 2 pores with diameters of about 20 µm. No cracks were detected in this specimen.

**Figure 12:**
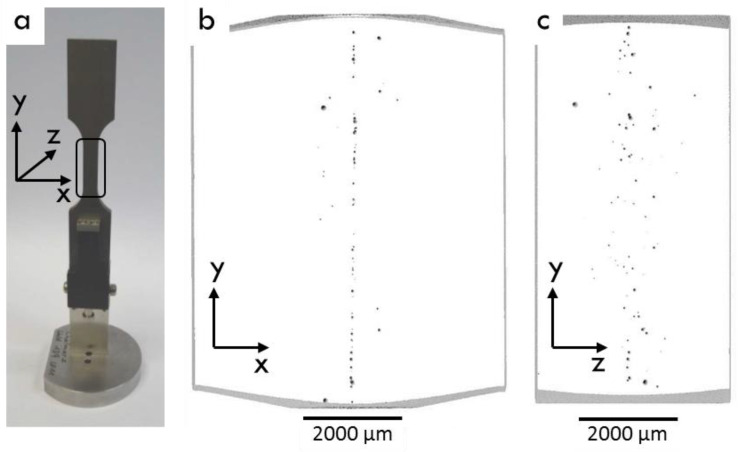
XRM 3D volume rendering showing distribution of porosity in a fatigue tested, but not fractured, LCF specimen produced by LBW: (**a**) overview of the LCF specimen, (**b**) view from top of the weld, x-y-plane, and (**c**) view from the side of the weld, y-z-plane.

In Figure 12, a 3D reconstruction from an XRM investigation of the LBW LCF specimens shown. The test was discontinued after 432,000 cycles with no failure. The XRM investigation showed that the LBW specimen contained continuous porosity along the centerline of the weld. In the z-direction of the sheet, the pores seemed to be more scattered but they were still mostly located close to the center of the weld. No porosity was observed close to the surface of the weld. The pore size along the centerline was normally less than 100 µm in diameter and the pores were mostly scattered. The largest pore detected in this sample was 113 µm in diameter. It was interesting to note that this number of pores and their distribution across the weld did not have any significant effect on the fatigue strength under the applied load conditions.

## 4. Discussion

Welded α + β titanium microstructures are complex. In all types of welds, the microstructure consists of fine α plates separated by thin layers of β phase. The thickness of the individual α plates in the EBW and LBW samples is on average below 1 µm, and in TIG and PAW, on average 1.2–1.3 µm. The microstructure of EBW and LBW welds show features of acicular α’, which is a result of martensitic transformation during fast cooling during welding. Upon post weld heat treatment at 704 °C, existing martensite has decomposed into α and β phases, but alpha laths have retained acicular morphology [24]. The resulting microstructure in EBW and LBW samples is a fine basketweave type of structure. TIG and PAW processes have larger heat input and slower cooling rates, which results in a mixture of martensitic and diffusionally transformed products. TIG welds have more of a colony type of microstructure, whereas PAW mainly contains the basketweave type of microstructure. As-welded samples were not available for investigation, so changes that occurred during post weld heat treatments are not possible to evaluate in detail.

The microhardness of the EBW, LBW and TIG were at the same level, whereas the PAW had slightly lower hardness. Typically, the maximum slip length defined by α colony size defines the hardness of α + β titanium alloys. Therefore, it was somewhat surprising to find such a high microhardness in the TIG-welded specimens, despite their relatively coarse microstructure. Oxygen and other interstitial elements, such as C and N, are well known to significantly increase the microhardness in titanium alloys [11]. In the present work, EPMA was used to evaluate whether a local increase in the amount of oxygen could have contributed to the unexpectedly high microhardness values, but no such difference between EBW and TIG welds was found. The yield strength and ultimate tensile strength of the welds were lower than in the base material, despite the higher hardness of the welds. The tensile strength of the TIG weld was lower than that of the EBW and LBW, even if their hardness was similar. The tensile tested weld specimens all fractured in the weld material. The fracture surfaces and etched side views of the fracture showed features of both intergranular and transgranural fracture. The tensile ductility depends on the strength difference between the prior-β grain boundary layer and the intragranular microstructure. In addition, the prior-β grain boundary length is related to tensile ductility, and to the prior-β grain size [11]. Therefore, the larger prior-β grain size found in the TIG welds could explain the lower ductility. TIG welds had the largest prior-β grain size, followed by PAW, that also had lowered ductility. LBW had the smallest prior-β grains, and also showed the highest ductility. Titanium alloys are very reactive at elevated temperatures and the pickup of interstitial elements such as O, N and C during welding has been suggested as a reason for lowered ductility [25]. As previously mentioned, EPMA measurements did not reveal elevated oxygen levels in TIG and EBW. In the TIG welding filler, material was used which may have had an effect on mechanical properties. Tensile tests in this study were performed in transverse direction to the weld. The weld joint is a composite with three different zones and is comprised of various microstructures. Thus, plastic strain could localize in a small area and thereby affect the measured elongation values. It has previously been shown that scattered porosity does not have an effect on static strength [6]. Here, porosity was found in a PAW and an LBW tensile test fracture surface. The specimens did not, however, show lower strength or ductility in comparison to the rest of the samples.

Fractography and XRM investigations showed that EBW and PAW produce welds with least porosity, and with the smallest pore size. The porosity found in EBW was smaller than 100 µm in diameter, and in PAW smaller than 200 µm in diameter. In PAW, a lack of fusion defect was also observed. TIG and LBW produced welds with more porosity, and of a larger size. In LBW, continuous porosity with small pore size was found along the centerline in the weld, with occasional large pores at the root side of the weld. In TIG, the porosity seemed to be scattered. In some TIG specimens several pores were observed in one fracture surface, but on the other hand XRM investigation of a discontinued LCF TIG specimen showed only two 20 µm size pores. LBW and TIG are known to be more susceptible for porosity than EBW and PAW [26], and the use of filler wire in TIG can increase the risk of porosity.

Total fatigue life is the sum of the cycles it takes to initiate a fatigue crack, and the number of cycles it takes for the crack to propagate to the critical size when final fracture occurs. At lower stress ranges the initiation part becomes larger, whereas at high stress range the crack initiation is fairly fast, and thus the major part of the total fatigue life is the crack propagation. Crack propagation is normally slower in coarse grained material, whereas crack initiation is inhibited by decreasing “grain size” (i.e., α colony size). Here, EBW and LBW welds with finer microstructure performed better than TIG and PAW welds with coarser microstructure. For LCF performance, the resistance to fatigue crack initiation is favored by a fine microstructure, and this seems to be more significant than the resistance to macrocrack growth rate favored by a coarser microstructure. Comparing EBW and PAW, the defects initiating a crack are in the same size range in both welds, but welds produced by EBW perform considerably better. In the welds produced by LBW, fractography and XRM showed several pores in the same size range that had not initiated fatigue cracks. Higher hardness, strength, and ductility, and the finer microstructure of EBW and LBW welds seem to be beneficial for LCF performance and make them less sensitive to porosity.

Under cyclic loading, there were a number of microcracks initiating, but only some of the microcracks kept growing and eventually caused failure. Numerous cracks were observed in individual specimens produced by LBW (Figure 9c) and TIG (Figure 11). It is often assumed that under high stress, as around a stress raiser such as a pore, cracks initiate almost immediately [27]. In the present work however, somewhat different findings were made. In the TIG specimen shown in Figure 11, three pores of similar size and location were found, but only one of the pores induced macrocracks that led to the final fracture. Pore 2 was found to initiate a microcrack and pore 3 had not initiated a detectable crack. This shows that crack initiation at small pores is difficult to predict, and may indicate that the time for a pore to initiate a crack is not negligible. Tammas-Williams et al. [19] found that surface defects are more harmful than a conventional fracture mechanics approach [18] would predict. The combination of defect size, location, aspect ratio, and proximity to other pores and the surface, provided a more accurate prediction of the most harmful pores.

As shown in Figure 7a, the majority of the samples tested at high stress ranges had a crack initiation at the surface of the sample (Figure 9d), which shows that cracks initiate easily at the specimen surface at high stress ranges. Most EBW and LBW specimens had a crack initiation with a facet at the surface of the sample. Facets in titanium alloys under cyclic loading can occur along basal planes, creating flat faceted features on fracture surface at a low-stress intensity range. Thousands of cycles can contribute to the formation of a facet [28]. Surface initiations with facets were observed in specimens tested at the highest stress ranges, and surface initiations were not found to occur at normalized stress ranges below 0.6. The specimens with crack initiation at a facet had the highest fatigue lives. At a high stress range, only large pores (>400 µm) were found to initiate cracks. Large pores were also found to be the most detrimental defect type for fatigue life. At 250 °C, small and internal pores were found to initiate cracks only at lower stress ranges where surface facet initiations did not occur. However, at room temperature, internal pores were found to initiate cracks in TIG and EBW samples at relatively high stress ranges (Figure 7b). At elevated temperatures, surface cracks become dominant more easily than internal cracks. Sarrazin-Bauduox et al. [29] showed that in Ti-64, the crack growth rate is higher in air than in a vacuum. The effect is more pronounced at elevated temperatures and for small cracks. Cracks initiated by internal defects grow in a quasi-vacuum. This is one reason why internal cracks grow slower than surface cracks and why surface cracks can become dominant more easily. At room temperature, the difference in crack growth rate is smaller and cracks initiated by internal pores have a bigger chance to become dominant, which may explain why LCF life seemed to be more sensitive to internal pores at room temperature than at elevated temperature. Åkerfeldt et al. [30] also suggested that increased ductility at elevated temperature might make LCF performance less sensitive to porosity.

## 5. Conclusions

Welding of titanium alloys is common practice in manufacturing industries today. However, the application of welding on more advanced fabrications and cyclic-loaded titanium alloy structures puts more emphasis on the importance of a complete understanding of the relationship between defects and microstructure, and their correlation with the mechanical integrity of welds. Therefore, the present work compares four of the most important welding processes for titanium alloys, with regard to unique welding process defect types/distribution, and weld microstructures and their relationship with mechanical properties. Based on the present work, the conclusions made are as follows:

Weld geometry, large pores, and pores close to surface were found to be the most detrimental to fatigue life.

At room temperature, LCF life was more sensitive to internal porosity than at elevated temperature.

Microstructure has a significant effect on fatigue performance as follows:

Pores initiated cracks in nearly all the samples in PAW and TIG welds, whereas in LBW and EBW most fatigue cracks initiated at the surface.

EBW and LBW showed superior fatigue performance over TIG and PAW.

LBW contained porosity but only large pores initiated fatigue cracks.

## Figures and Tables

**Table 1:** Chemical compositions of the Ti-64 sheet and filler wire materials in wt.%.

	Al	V	Fe	C	O	N	H	Y	Ti
**Sheet**	6.12	3.81	0.17	0.006	0.12	0.005	<0.001	<0.005	Bal.
**Filler wire**	5.50–6.75	3.50–4.50	<0.22	<0.05	<0.18	<0.03	<0.015	<0.005	Bal.

**Table 2:** Measured microstructural features in the different welds.

	TIG	PAW	EBW	LBW
**FZ width top/bottom**	12.3/7.5 mm	7.3/5.8 mm	3/3 mm	3/1.8 mm
**HAZ width**	3.2 mm	3.0 mm	1.8 mm	1.6 mm
**Maximum prior-β grain size**	3 mm	2 mm	1.5 mm	1 mm
**GB-α**	Approx. 1 µm/continuous	Approx. 1 µm/continuous	Thin/intermitted	Thin/intermitted
**α lath spacing**	1.2–1.3 µm	1.2–1.3 µm	0.8–1 µm	0.8–1 µm
**HV0.5**	342 ± 12.9	325 ± 15.4	335 ± 6.9	339 ± 8.6

**Table 3:** Tensile properties of the welds at room temperature (RT) and at 250 °C.

	Yield Strength—RT (Mpa)	Yield Strength—250 (MPa)	Tensile Strength—RT (MPa)	Tensile Strength—250 (MPa)	Tensile Elongation—RT (%)	Tensile Elongation—250 (%)	Reduction of Area—RT (%)	Reduction of Area—250 (%)
**BM**	975	735	1021	800	16.4	17.8	44	62
**TIG**	871	596	958	749	7	14	10	38
**PAW**	856	590	954	729	9	14	17	43
**EBW**	940	689	1014	795	10	15	16	42
**LBW**	931	677	995	773	10	12	23	39

**Table 4:** Summary of results from fractography on samples tested at 250 °C. The number of tested specimens, number of surface initiations, initiations at a pore, and the size distribution of all pores found on the fracture surfaces.

Size [µm]	TIG	PAW	EBW	LBW
Samples tested/Eligible for fractography	26/16	19/8	40/28	23/12
Surface initiation	3	2	24	9
Initiation at pore	13	6	4	3
0–100	3	3	4	15
100–200	10	3	-	3
200–300	6	-	-	-
300–400	4	-	-	-
400–500	1	-	-	-
600–700	-	LOF	-	3

## Data Availability

Data are contained within the article.
